# 3D human liver tissue from pluripotent stem cells displays stable phenotype in vitro and supports compromised liver function in vivo

**DOI:** 10.1007/s00204-018-2280-2

**Published:** 2018-08-28

**Authors:** Hassan Rashidi, Nguyet-Thin Luu, Salamah M. Alwahsh, Maaria Ginai, Sharmin Alhaque, Hua Dong, Rute A. Tomaz, Bertrand Vernay, Vasanthy Vigneswara, John M. Hallett, Anil Chandrashekran, Anil Dhawan, Ludovic Vallier, Mark Bradley, Anthony Callanan, Stuart J. Forbes, Philip N. Newsome, David C. Hay

**Affiliations:** 1 MRC Centre for Regenerative Medicine, University of Edinburgh, Edinburgh, EH16 4UU UK; 2 Centre for Liver Research, Institute of Immunology and Immunotherapy and National Institute for Health Research Biomedical Research Centre at University Hospitals Birmingham NHS Foundation Trust and the University of Birmingham, Birmingham, UK; 3 Liver Unit, University Hospitals Birmingham NHS Foundation Trust, Birmingham, UK; 4 Institute of Bioengineering, The University of Edinburgh, King’s Buildings, Edinburgh, EH9 3DW UK; 5 School of Chemistry, University of Edinburgh, Kings Buildings, EH9 3FJ Edinburgh, UK; 6 Anne McLaren Laboratory, Wellcome Trust-MRC Stem Cell Institute, University of Cambridge, Cambridge, CB2 0SZ UK; 7 Child Health Clinical Academic Group, MRC Centre for Transplantation, King’s College London, London, UK

**Keywords:** Liver tissue, Pluripotent stem cell, Stable cell phenotype, Implantable liver graft, Interdisciplinary research

## Abstract

Liver disease is an escalating global health issue. While liver transplantation is an effective mode of therapy, patient mortality has increased due to the shortage of donor organs. Developing renewable sources of human liver tissue is therefore attractive. Pluripotent stem cell-derived liver tissue represents a potential alternative to cadaver derived hepatocytes and whole organ transplant. At present, two-dimensional differentiation procedures deliver tissue lacking certain functions and long-term stability. Efforts to overcome these limiting factors have led to the building of three-dimensional (3D) cellular aggregates. Although enabling for the field, their widespread application is limited due to their reliance on variable biological components. Our studies focused on the development of 3D liver tissue under defined conditions. In vitro generated 3D tissues exhibited stable phenotype for over 1 year in culture, providing an attractive resource for long-term in vitro studies. Moreover, 3D derived tissue provided critical liver support in two animal models, including immunocompetent recipients. Therefore, we believe that our study provides stable human tissue to better model liver biology ‘in the dish’, and in the future may permit the support of compromised liver function in humans.

**Electronic supplementary material:**

The online version of this article (10.1007/s00204-018-2280-2) contains supplementary material, which is available to authorized users.

## Introduction

The liver performs a wide range of functions essential for body function. Significant loss of liver function therefore has serious consequences for human health (Ebrahimkhani et al. 2014). In the UK, liver disease is the fifth biggest killer, and unlike the top four, the incidence is increasing. Orthotopic liver transplantation (OLT) is the most effective treatment for acute or end-stage liver failure. However, due to the shortage of organ donors, and complications associated with lifelong immunosuppression, there is an impetus to develop alternative therapies.

While donor hepatocytes have been used to successfully treat metabolic liver disease (Alwahsh et al. 2018), the graft is eventually lost. This is further compounded by the scarcity of human hepatocytes. Therefore, we and others have proposed that pluripotent stem cells (PSCs) are a credible alternative with which to generate a stable source of quality assured human liver tissue for applied medicine. PSCs can self-renew and differentiate to all cells types in the body, promising an unlimited supply of human tissue for application. To this end, several protocols have been developed to efficiently generate hepatocyte-like cells (HLCs), mainly employing two-dimensional differentiation systems (Hay et al. 2007, 2008a, b; Agarwal et al. 2008; Sullivan et al. 2010; Hannan et al. 2013; Loh et al. 2014). Despite recent improvements (Takayama et al. 2013; Cameron et al. 2015; Wang et al. 2017), 2D-derived HLCs exhibit foetal features and unstable phenotype in vitro, limiting their clinical application.

To improve hepatocyte stability, three-dimensional (3D) approaches have been explored to generate human liver tissue (Szkolnicka and Hay 2016). Those processes employ matrix or scaffold-driven formation of 3D aggregates with or without inclusion of other cell types (Gieseck et al. 2014; Takebe et al. 2014; Camp et al. 2017). These models have been important to further our understanding of human development and physiology. However, they were manufactured using components which suffer from batch variation and this limits widespread application of the technology.

In these studies, we addressed these issues describing a scalable and defined differentiation process to generate 3D human liver tissue. Notably, stem cell derived liver tissue displayed modest liver function for more than 365 days in culture. During differentiation, cell organisation became evident, correlating with stable function, reduced proliferation and loss of foetal protein secretion. Stem cell derived liver tissue was also transplanted in vivo providing critical liver support in immune-competent or deficient recipients with compromised liver function.

In conclusion, our study demonstrates that functional human liver tissue, derived from pluripotent stem cells, can be stably cultured in vitro for long periods of time and provide liver support in vivo. We believe this resource provides promise for in vitro applications, such as repeated dosing and disease modelling, and in the future may serve as a source of tissue for the clinic.

## Experimental procedures

### Maintenance of human PSCs

hPSC lines, including two embryonic stem cell lines (H9, Man12) and iPSC lines (FSPS13B and P106) were cultured on laminin 521 (BioLamina) coated plates in serum-free mTeSR^TM^1 medium (STEMCELL Technologies) as previously described (Cameron et al. 2015). The cell lines were monitored regularly for mycoplasma infection and were propagated in antibiotic free medium.

### Formation of self-aggregated PSCs spheroids

Agarose microplates were manufactured in 256-well format using the3D Petri Dish^®^ mould (Sigma Aldrich) following the manufacturer instructions and transferred to 12-well plates (Corning). hPSCs scaled up on laminin-coated plasticware, were incubated with 1 ml of Gentle Dissociation Buffer (STEMCELL Technologies) for 7–10 min at 37 C. Following this, single cell suspensions were prepared by pipetting the buffer up and down gently. The cell suspension was centrifuged at 0.2 rcf for 5 min and resuspended in mTeSR1 supplemented with 10 µM Y-27,632 (Calbiochem) at a density of 2.0 × 10^6^ live cells/ml. The agarose microplates were seeded by transferring 190 µl of cell suspension. After 2–3 h, 1 ml mTeSR1 supplemented with 10 µM Y-27,632 was gently added to each well of 12-well plate and incubated overnight.

### Hepatic induction of self-aggregated PSCs spheroids

Differentiation was initiated replacing mTeSR^TM^1 with endoderm differentiation medium: RPMI 1640 containing 1 × B27 (Life Technologies), 100 ng/mL Activin A (PeproTech), and 50 ng/mLWnt3a (R&D Systems). The medium was changed every 24 h for 72 h. On day 4, endoderm differentiation medium was replaced with hepatoblast differentiation medium, and this was renewed every second day for a further 5 days. The medium consisted of knockout (KO)-DMEM (Life Technologies), knockout serum replacement (KOSR - Life Technologies), 0.5% Glutamax (Life Technologies), 1% non-essential amino acids (Life Technologies), 0.2% b-mercaptoethanol (Life Technologies), and 1% DMSO (Sigma). On day 9, differentiating cells were cultured in the hepatocyte maturation medium HepatoZYME (Life Technologies) containing 1% Glutamax (Life Technologies), supplemented with 10 ng/ml hepatocyte growth factor (HGF; PeproTech) and 20 ng/ml oncostatin m (PeproTech) as described previously (Cameron et al. 2015; Wang et al. 2017). On day 21, cells were cultured in maintenance medium consisted of William’s E media (Life Technologies), supplemented with 10 ng/ml EGF (R&D Systems), 10 ng/ml VEGF (R&D Systems), 10 ng/ml HGF (PeproTech), 10 ng/ml bFGF (PeproTech), 10% KOSR, 1% Glutamax, and 1% penicillin–streptomycin (Thermo Fisher Scientific) for the rest of study.

### Histology and immunofluorescence

3D spheroids were fixed in ice-cold methanol for 30 min., washed in PBS, and embedded in agarose. Agarose-embedded spheroids were embedded in paraffin and 4 µm sections were obtained. Antigen retrieval was performed by heating dewaxed and rehydrated sections in 1 × Tris–EDTA buffer solution for 15 min in microwave. Washed slides were used for subsequent staining.

Paraffin-embedded 4-µm liver sections were stained with Eosin and Hematoxylin and mounted in Pertex before microscopy. To assess glycogen storage, sections were stained by periodic acid-Schiff (PAS, Sigma) following the manufacturer’s instructions. Brightfield images were taken using a Nikon Eclipse e600 microscope equipped with a Retiga 2000R camera (Q-Imaging) and Image-Pro Premier software.

To stain sectioned spheroids, stem cell derived tissue was blocked with 10% BSA in PBS-tween (PBST) and incubated with primary antibody overnight at 4 °C, and detected using species-specific fluorescent-conjugated secondary antibody (Alexa Flour 488/Alexa Flour 568; Invitrogen). Sections were counterstained with DAPI (4′,6-diamidino-2-phenylindole) and mounted with Fluoromount-G (SouthernBiotech) before microscopy.

An extended staining protocol was developed and optimised to stain whole mount 3D Heps. Briefly, MeOH fixed spheroids were washed and rehydrated in PBS. Following overnight blocking in 10% BSA in PBST, the spheroids were incubated with primary antibody at desired concentration (Supplementary Table 1) with gentle agitation at 4 °C overnight followed by 8 washes with 0.1% PBST (each wash 1 h) under gentle agitation at room temperature (RT). Secondary antibodies were incubated at 4 °C overnight and washed as described above (Supplementary Table 2). The nucleus was counterstained with Draq5 (Life Technologies) before images were acquired on a confocal microscope (Leica SP5).

### qRT-PCR

RNA was extracted from 3D Heps using an RNeasy Mini RNA Extraction kit (Qiagen). RNA quantity and quality were assessed using a Nanodrop system. Following this, cDNA was amplified using the RT^2^ First Strand Kit (Qiagen) following the manufacturer’s instruction. qPCR was performed with TaqMan Fast Advance Mastermix and primer pairs listed in Supplementary Table 3 and analysed using a Roche LightCycler 480 real-time PCR system. Gene expression was normalised to glyceraldehyde 3-phosphate dehydrogenase (GAPDH) and expressed as relative expression over 3D aggregate on day 0 of differentiation as control sample. qPCR was performed in triplicate and data analysis was performed using Roche LightCycler 480 software (version 1.5).

### Hepatocyte phenotyping

To measure Cyp3A activity, 50 µM of Luciferin-PFBE substrate (Promega) was incubated with cells in HepatoZYME medium supplemented with 10 ng/ml HGF. Cytochrome P450 activity was measured 24 h later using the P450-Glo assay kit (Promega) according to manufacturer’s instruction. To measure AFP and ALB secretion, the culture medium was collected after 24 h and quantified using commercial available ELISA kits (Alpha Diagnostics International). Data were normalised with the total protein content measured using bicinchonic acid (BCA) assay (Thermo Fisher Scientific).

For the intraperitoneal transplant study, human ALB level was measured by ELISA (MULTI-SPOT® 96 4-Spot Custom Human ALB Singleplex, Maryland, USA), in serum collected from 50% PHx after 4 weeks post vehicle or 2 × 10^6^ hepatospheres transplantation. Stored serum samples were thawed at room temperature and tested for ALB concentration which is quoted as pg/mL.

### Scaffold production

Non-woven Polycaprolactone (PCL; Sigma Aldrich, UK) scaffolds were produced using the EC-DIG electrospinning platform (IME Technologies, Netherlands). Low concentration (8% wt/v) and high concentration (14% wt/v) PCL solutions were generated by dissolving PCL pellets on a rolling mixer overnight in Hexafluoroisopropanol (HFIP; Manchester Organics, UK) or Chloroform/ethanol (5:1) (Sigma Aldrich, UK), respectively. The solutions were then used to generate electrospun scaffolds with small and large fibre diameters. To fabricate small fibre with diameter of 1.38 µm ± 0.16 µm, electrospinning experiments were carried out with low concentration (8% wt/v) PCL solution and a 26G needle. The feeding rate of the solution was 2.0 mL/h. The voltage was between 14 and 16 kV. And the distance between the needle tip and the collecting substrate was 15 cm with mandrel speed of 250 rpm. To generate large fibre with diameter of 6.68 µm ± 1.18 µm, high concentration (14% wt/v) PCL solutions was fed at 7.0 ml/h. The distance between the needle tip and the collecting substrate was 18 cm and the voltage was 20 kv. The size of needle and mandrel speed was similar to small fibre.

To fabricate hexagonal electrospun scaffolds, polycaprolactone (Scientific Polymer, US, Mw = 100,000) was dissolved in a mixed solvent of DMF and THF (1/1, w/w) with a final polymer concentration of 14% (w/w). Electrospinning experiments were carried out on a typical electrospinning setup (Spraybase, Ireland), with a 22G needle. The feeding rate of the solution was 0.3 mL/h. The voltage was between 6 and 8 kV. And the distance between the needle tip and the collecting substrate was 7 cm. Room temperature was controlled at 20 °C, and the relative humidity was around 40%.

### Scanning electron microscopy

Scanning electron microscopy (SEM) (Hitachi S-4700; Hitachi, Japan) was used to image the electrospun mats. Samples were sputter coated with gold/palladium using the E5200 automatic sputter coater (Polaron; Quorum Technologies, UK).

### Intraperitoneal transplantation

Both male (30–36 g) and female (23–27 g) C57BL6/J, Rag2^−/−^IL2rg^−/−^ mice that lack lymphocytes (B and T) and natural killer cells were used. Animals were housed in a specific pathogen-free environment and kept under standard conditions with a 14 h day/10 h night cycle and had a free access to food and water. All animal experiments were performed under procedural guidelines, severity protocols, and with ethical permission from the University of Edinburgh Animal Welfare and Ethical Review Body and the UK Home Office.

Liver injury was induced in immunocompromised (Rag2^−/−^IL2rg^−/−^) mice by commonly used Three Knots methods for partial hepatectomy (PHx). 50% PHx, the left lobe and the left part of the median lobe were excised. Gallbladder was preserved in this surgical procedure. Mice received 200 µL HepatoZYME (vehicle) or 2 × 10^6^ hepatospheres that were loaded intraperitoneally. The abdominal incision was closed and animals were monitored closely until recovery from anaesthesia. Body weights of the rodents were measured 10 days before starting the experiment and every day after transplantation. Four weeks after human cell transplantation, the recipient mice were euthanized according to UK Home Office regulations. Blood was collected by cardiac puncture and centrifuged to collect serum. Livers were harvested and fixed in 4% buffered-formaldehyde for 24 h and were then stored in paraffin. Animals reaching experimental severity protocol boundaries were excluded from analysis; otherwise, all animals were included. Control group (no surgery, *n* = 5) was used to determine the baseline of liver function and injury tests.

### Subcutaneous transplantation

Immune-deficient, fumarylacetate hydrolase (Fah^−/−^), Rag2^−/−^ and Il2rg^−/−^ animals on the NOD-strain background (FRGN) were originally obtained from Yecuris. The immune-normal, Rag2^+/+^/Il2rg^+/+^, with Fah knockout mice (FAH) have been generated and maintained by the Newsome group at Birmingham. Animals were maintained and housed under conventional conditions in the Biomedical Service Unit at the University of Birmingham. They were at constant temperature and humidity in a 12-h controlled light–dark cycle and were used in accordance with Home Office guideline. Surgery was performed efficiently and strictly according to approved protocol for induction of liver injury under project licence (P3CDEF650), with maximal care to minimize suffering. They regained consciousness within 5–10 min after surgery and given pain-relief incorporated in the soft food for maximal comfort.

Animals were aged 8–12 weeks at the start of the experiments. Following isoflurane anaesthesia a small incision (~ 1 cm) was made on the left lower back of the animal. The scaffold (10 mm in size) was carefully placed onto the body tissue facing downwards. After suture the animals were allowed to recover and kept on the medication: NTBC to prevent liver damage for a further 3 days to allow the implant to settle. Following this NTBC support was replaced with normal drinking water which would induce liver injury due to the accumulation of toxic metabolites. Animal wellbeing was monitored every other day for further 14 days.

### Histopathological sections

Samples were fixed for at least 24 h in 10% neutral buffered formalin solution (pH7.4) at room temperature (RT). Tissue was embedded in paraffin and sectioned at 4 µm. Prior to staining, sections were dewaxed in xylene and rehydrated using graded industrial denatured alcohol (IDA). Tissue sections were stained with standard histological Harris’s Haematoxylin and Eosin (H&E) (Leica, German). All sections were rehydrated in graded IDA, cleared in xylene and were mounted with DPX (leica Biosystems,UK); cover slipped and images were captured with Zeiss Axio ScanZ1.

### Serum level of liver damage biomarkers and Human albumin ELISA

Blood samples were withdrawn by cardiac puncture during isofluorane anesthesia and allowed to clot at room temperature for at least 30 min. After centrifugation serum was collected and stored at − 80 °C until use. The mouse liver biomarkers: ALT, AST, bilirubin, and ALB, were measured at the Clinical Biochemistry Laboratory of the Women’s Hospital Birmingham (Birmingham, UK). Human ALB in the mouse serum samples was measured using a Human Albumin ELISA Quantitation Kit (Abcam,UK) according to the manufacturer’s instructions.

### Statistics

Data were analysed by GraphPad Prism (version 7). Student’s *t* test or Mann–Whitney tests were used to determine the difference, which was set at *P* < 0.05. We did not use statistical methods to predetermine sample size, there was no randomization designed in the experiments, and the studies were not blinded. Number of animals = 3–20 per group from 12 independent experiments, significance was determined by One-way analysis of ANOVA Bonferroni, Mann Whitney test or by Tukey post hoc, as suitable. Data are represented as mean ± SEM or median where appropriated, *p < 0.05, **p < 0.01, ***p < 0.001. ****p < 0.0001; *comparison of SC-HLC verse SC + HLC during NTBC withdraw. ^+^ is the comparison of control NTBC to the SC-HLC-NTBC group.

## Results

### Building stable and defined 3D human liver tissue in vitro

**Fig. 1:**
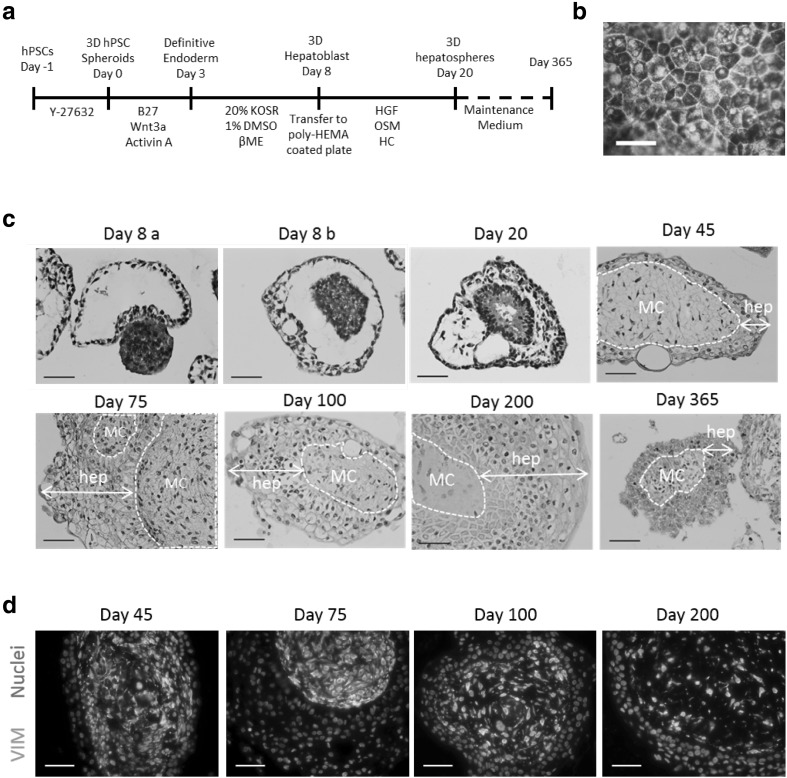
Generation of scalable, defined and stable 3D hepatospheres from hPSCs. **a** A stepwise defined protocol for the generation of 3D Heps. **b** Brightfield image of 3D Heps at day 365 of differentiation. Scale bar, 50 µm. **c** H&E staining of 3D spheres at various time points. A central mesenchymal core (MC) was surrounded by several layers of hepatocytes (hep). Scale bar, 50 µm. **d** Expression of vimentin (VIM) was detected in cells located in MC. Nuclei were stained with DAPI. Scale bar, 50 µm

Aggregated human embryonic stem cells (hESCs) and induced pluripotent stem cells (hiPSCs) were differentiated toward the hepatocyte lineage in suspension or agarose multiwell plates using a defined procedure (Fig. 1a and Supplementary Fig. 1). To produce homogenous three-dimensional spheres (3D Heps) of defined size, the agarose multi-well plate technology was routinely employed (Supplementary Fig. 1). During cellular differentiation, 3D Heps exhibited typically hepatic morphology with distinctive cell borders which was maintained until termination of cell culture at d365 (Fig. 1b). Haematoxylin and Eosin staining indicated that the outer layer was 2–3 cells in depth with a dense core that was expelled in most spheroids between days 8 and 10 to form cystic 3D Heps which could be harvested (Day 8a; Fig. 1c). By day 20, structural reorganisation was observed (Fig. 1c), a process that continued until day 45, when two distinct compartments were observed. The outer layer was composed of hepatocyte-like cells with mesenchymal vimentin positive cells in the core (Fig. 1d). At these stages of differentiation cell proliferation decreased (Supplementary Fig. 2a) and 3D Heps acquired the ability to store glycogen (Supplementary Fig. 2b).

### Tracking endoderm differentiation from PSCs

**Fig. 2:**
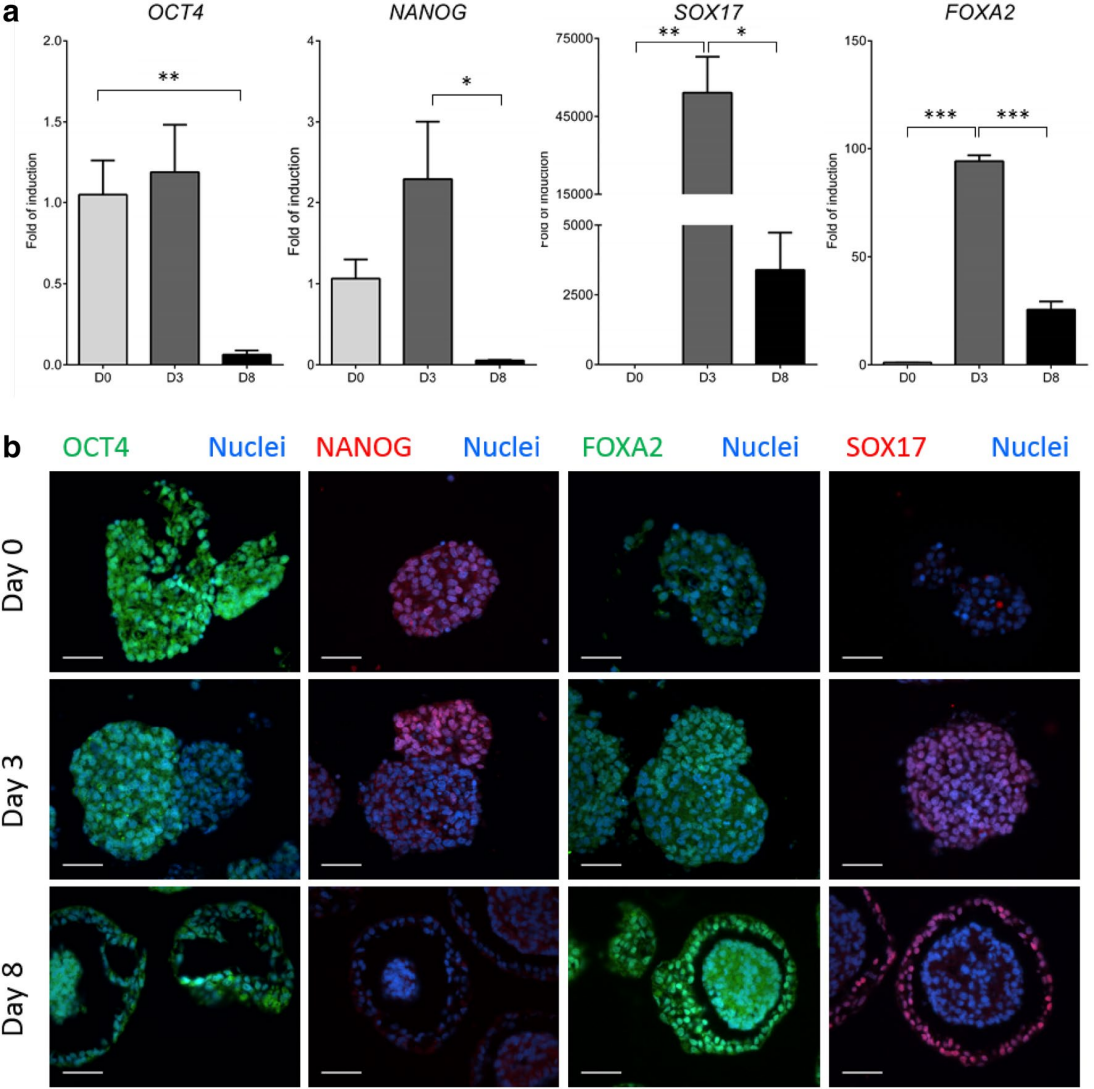
Evaluation of pluripotent and definitive endoderm markers. **a** Downregulation of *OCT4* and *NANOG* expression by d8 which was accompanied by upregulation of *SOX17* and *FOXA2* as markers of definite endoderm by d3 of differentiation. **b** Expression of Oct4 and Nanog was reduced during differentiation. In contrast, the expression of Sox17 and FoxA2 increased during differentiation. *n* = 3 per group, significance was determined One-way ANOVA Tukey post hoc test. Data are represented as mean ± SEM, *p < 0.05, **p < 0.01, ***p < 0.001. Nuclei were stained with DAPI. Scale bar is 50 µm

**Fig. 3:**
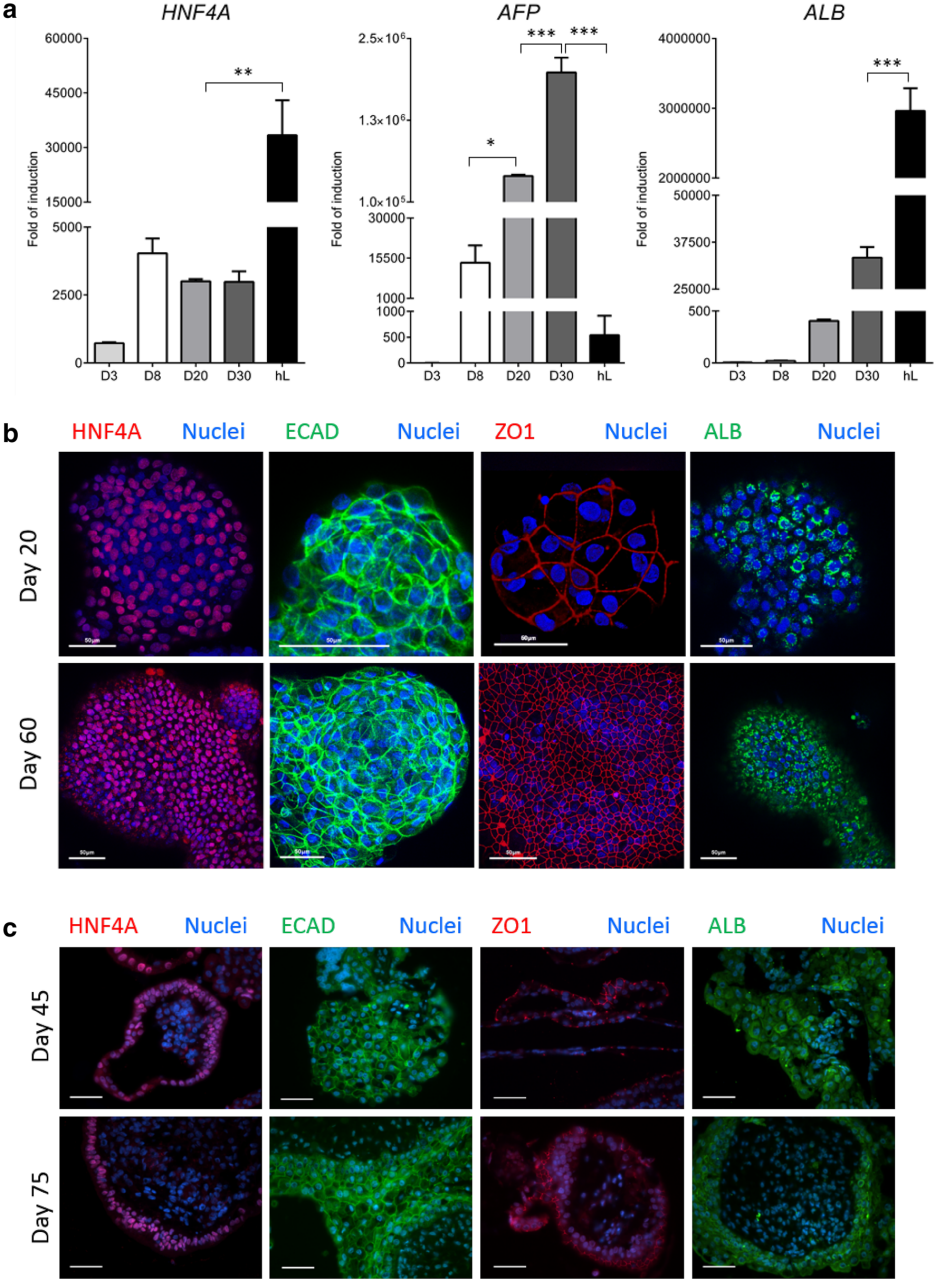
Evaluation of hepatic markers at gene and protein level. **a** Increased expression of *HNF4A, AFP* and *ALB* during cell differentiation and compared to human liver (HL). **b** Confocal microscopy of 3D Heps for Hnf4A, ECad, ZO1 and Alb. **c** Expression of hepatic markers was limited to cells within the periphery. *n* = 3 per group, significance was determined by one-way ANOVA Tukey post hoc test. Data are represented as mean ± SEM, *p < 0.05, **p < 0.01, ***p < 0.001. Nuclei were stained with DAPI. Scale bar is 50 µm

**Fig. 4:**
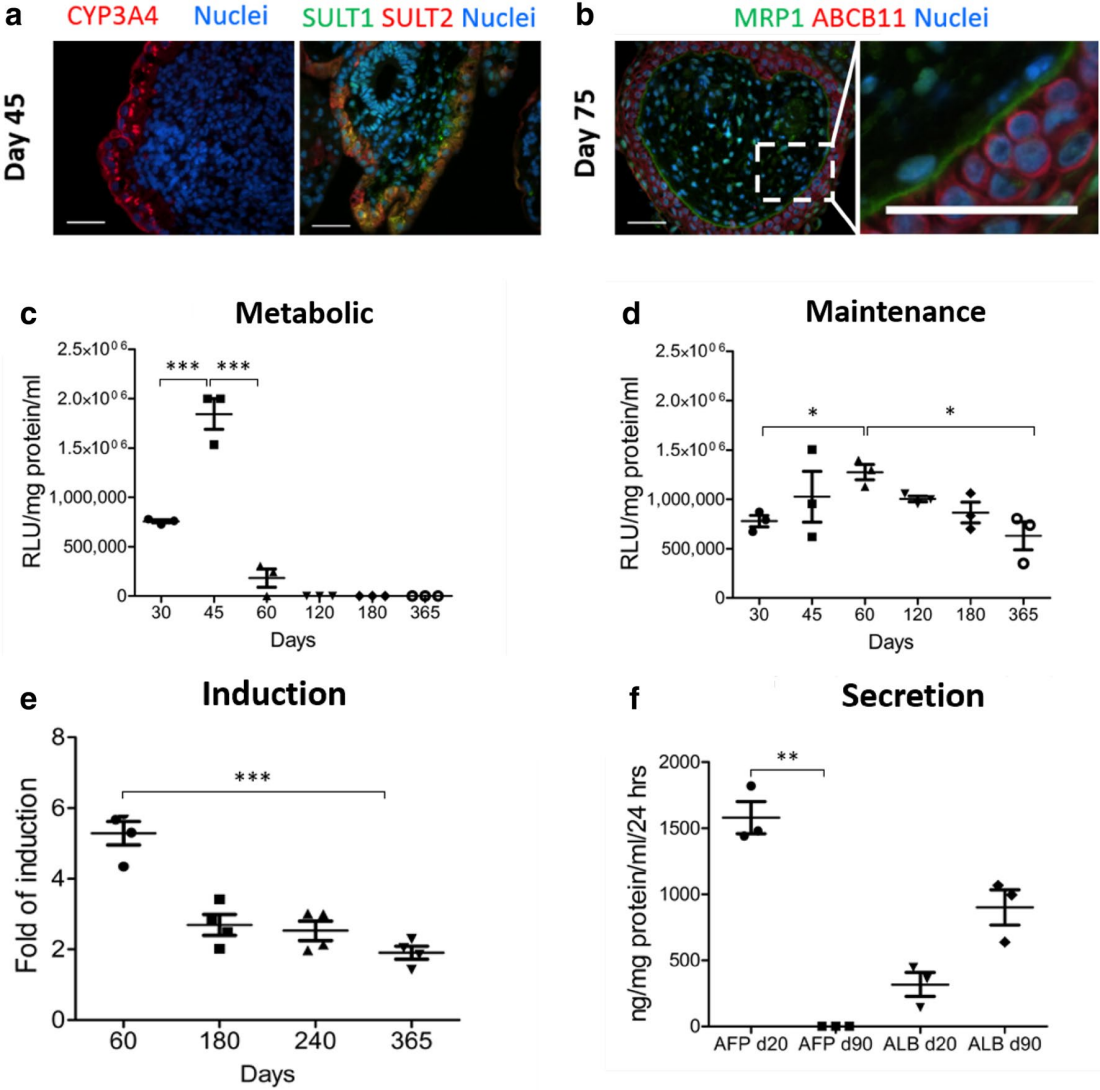
Functional analysis of 3D Heps. **a** Expression of Phase I (Cyp3A4) and Phase II (SULT1 & SULT2) markers in 3D Heps. **b** Expression and localisation of drug transporters (MRP1 and ABCB11) in d45 3D Heps. **c** Cyp3A activity of differentiated 3D Heps maintained in HepatoZYME. **d** Long-term Cyp3A activity of differentiated 3D Heps maintained in maintenance medium. **e** 3D Heps remain drug inducible for 365 days in vitro. **f** Secretion of AFP and ALB in 3D Heps at days 20 and 90

During cellular differentiation, we examined pluripotent and definitive endoderm gene expression from d0 to d8. During differentiation, 3D aggregates down-regulated the pluripotent transcripts, *OCT4* and *NANOG. SOX17* and *FOXA2* levels increased as the cells differentiated toward definitive endoderm (Fig. 2a). These data were confirmed by immunostaining of sectioned spheroids (Fig. 2b). Spheroid commitment to the hepatic lineage was supported by increased transcript levels for hepatic nuclear factor 4 alpha (*HNF4A*), alpha fetoprotein (*AFP*) and albumin (*ALB*) (Fig. 3a). These data were further supported by whole mount immunostaining of 3D Heps. 3D Heps at d20 expressed the tight junction marker ZO1, the liver enriched transcription factor HNF4A, ALB and an epithelial marker, E-Cadherin (ECAD) (Fig. 3b). 3D Hep protein expression was examined 40 days later demonstrating stable expression of HNF4A, ALB, ECAD and ZO1 (Fig. 3b). To examine 3D Hep structure, we performed sectioning followed by immunostaining for ALB, HNF4A, AFP, ECAD, alpha 1 anti-trypsin (A1AT), PROX1 and ZO1 at two time points. Hepatic marker expression was limited to cells on the periphery and absent from the sphere’s core (Fig. 3c, Supplementary Fig. 3). Similarly, the expression of phase I and II proteins: Cyp3A4 (red) (Fig. 4a) and SULT1 (red) and SULT2 (green) (Fig. 4a) was restricted to the outer layer. A similar pattern was observed for the cell membrane transporters, multi-drug resistant protein 1 (MRP1, green), and ATP-binding cassette sub-family B member 11 (ABCB11, red) (Fig. 4b).

### 3D Heps are active for up to 1 year in vitro

Post-gene expression analysis, we determined the functional capacity of stem cell-derived liver tissue. To build a stable system, we developed a maintenance medium that supported long-term culture of 3D liver tissue in vitro. Our previous attempts for long-term culture using metabolic medium led to sphere disaggregation, reduced Cyp3A activity and cell death by d60 in vitro (Fig. 4c), whereas the 3D maintenance medium permitted the culture of 3D Heps for 365 days with respectable Cyp3A activity (Fig. 4d). Mature metabolic activity was corroborated by drug inducible Cyp3A activity (Fig. 4e) and by the loss of AFP secretion and maintenance of ALB secretion (Fig. 4f).

### 3D Heps can be generated from hESC and hiPSC lines using the same methodology

To determine the reproducibility of our protocol, parallel differentiation of two hESC (H9 & Man12) and two hiPSC (FSPS13B and P106) lines was performed. The efficiency of the process was examined by analysing protein secretion and metabolic activity at d20. 3D Heps were derived successfully from all four tested lines with varying levels of function. Among tested lines, Man12 had secreted 3.4 µg of AFP per mg protein into the culture media in 24 h which was significantly higher than the other three lines (Supplementary Fig. 4a). Similarly, Man12 secreted 800 ng ALB into the culture media which was the highest among tested lines (Supplementary Fig. 4b). Cyp1A2 activity was notably higher in iPSC 3D Heps compared to hESC-derived 3D Heps (Supplementary Fig. 4c). Similar levels of Cyp3A activity were detected in all 3D Heps, except for Man12 which displayed lower levels (Supplementary Fig. 4d), consistent with a previous study (Cameron et al. 2015).

### 3D Heps improve rodent recovery following partial hepatectomy

Stem cell-derived 3D Heps were transplanted intraperitoneally immediately following 50% partial hepatectomy (PHx) into C57BL6/J, Rag2^−/−^IL2rg^−/−^mice. Mice which received vehicle control lost about 8–10% of their initial body weights and started to regain weight two weeks after surgery. In contrast, mice that received 3D Heps regained weight within 4 days of surgery and recovered their initial body weight after 14 days (Fig. Supplementary 5a). These observations were supported by secretion of human albumin in 3D Hep recipient mice (Supplementary Fig. 5b) and a reduction in aspartate aminotranferase (AST), a marker of hepatocellular injury (Supplementary Fig. 5c). In both treatment groups circulating bilirubin levels remained unaltered (Supplementary Fig. 5d) While these experiments provided proof of concept, we decided to take a less invasive approach to provide mammalian liver support, acknowledging that this would be the most likely treatment option for liver disease in patients not eligible for cell based therapy or a liver transplant.

### Sub-cutaneous implant of 3D Heps to support failing liver function in vivo

**Fig. 5:**
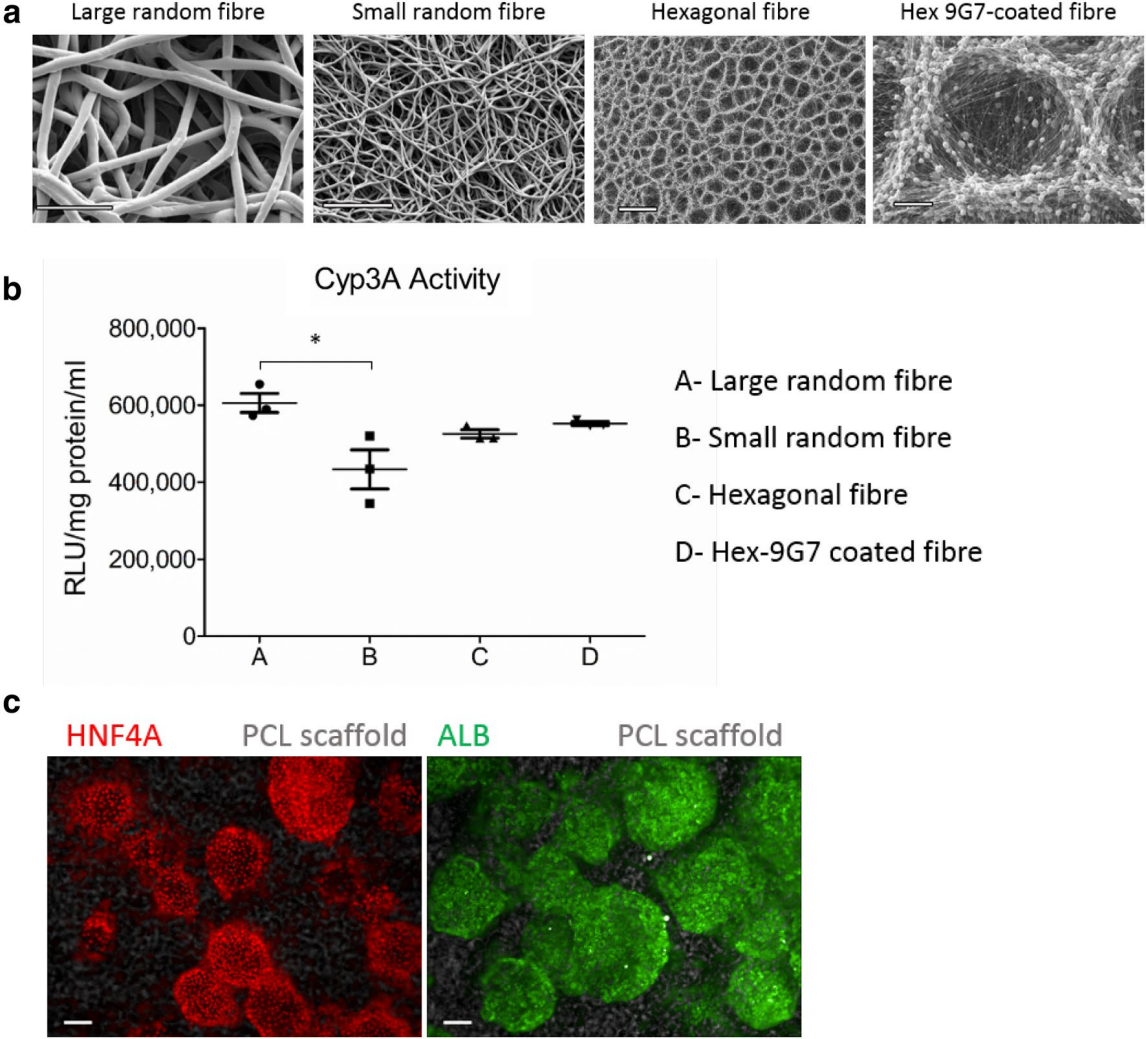
Development and characterisation of a sub-cutaneous implant. **a** Fabrication of electrospun PCL scaffolds with four different geometries. **b** 3D Heps remained metabolically functional 16 days post seeding on electrospun scaffolds with peak activity on large random fibre. **c** 3D Heps maintained expression of key markers including HNF4A and ALB following seeding on the large fibre scaffold. *n* = 3 per group; significance was determined by one-way ANOVA Tukey post hoc test. Data are represented as mean ± SEM, *p < 0.05. Scale bar is 50 µm

Working with materials chemists and engineers, we identified FDA approved materials suitable for human use. Electrospun scaffolds were prepared from polycaprolactone (PCL) with four different geometries and one coated with polyacrylate (polymer 9G7). H9-derived 3D Heps were seeded on the PCL scaffold at d14 of differentiation and attached to the fibres (Fig. 5a). 16 days post replating, 3D Heps displayed Cyp3A P450 function (Fig. 5b) and maintained expression of Hnf4a and Alb (Fig. 5c). The fabrication of large PCL fibres was most reproducible and was used for the sub-cutaneous implant experiments.

**Fig. 6:**
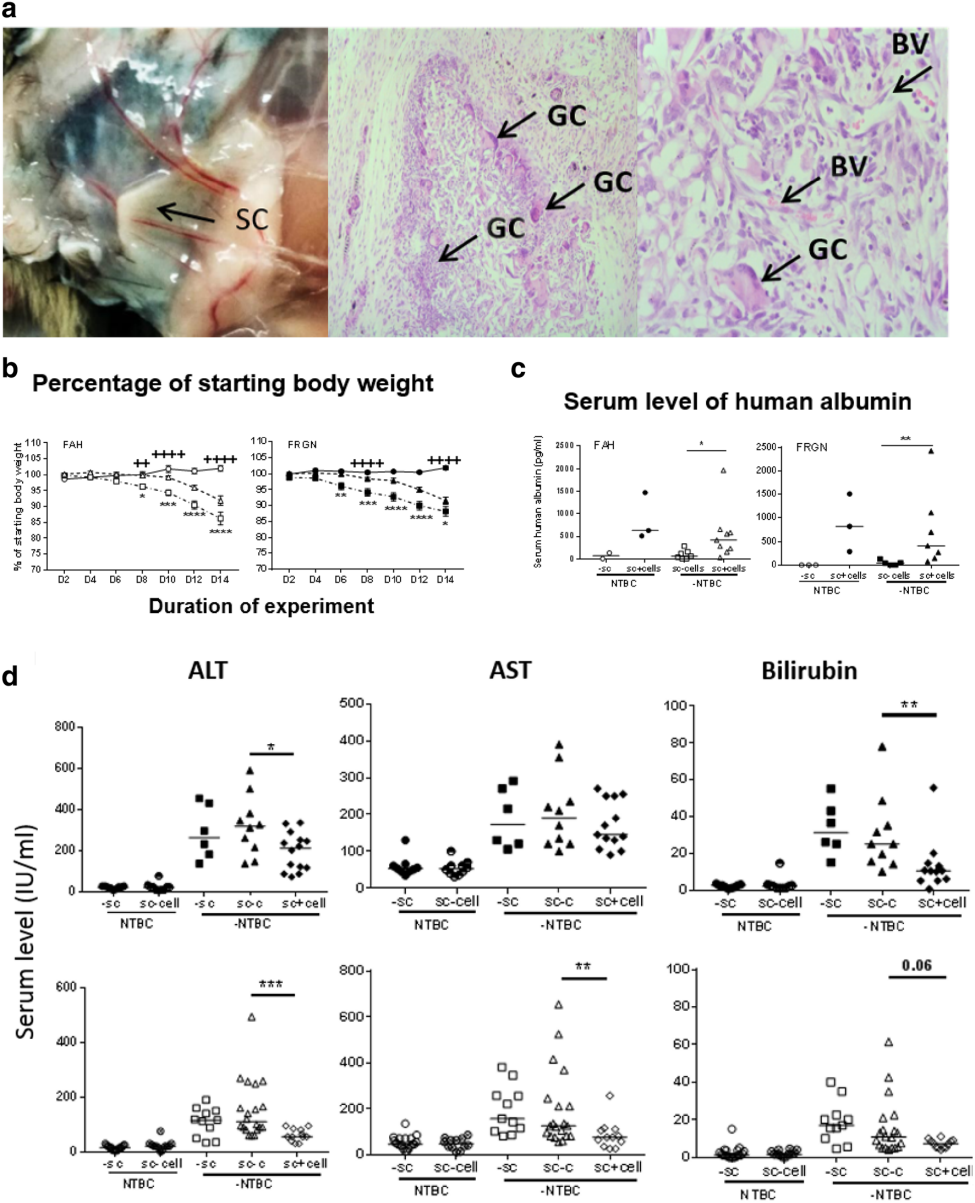

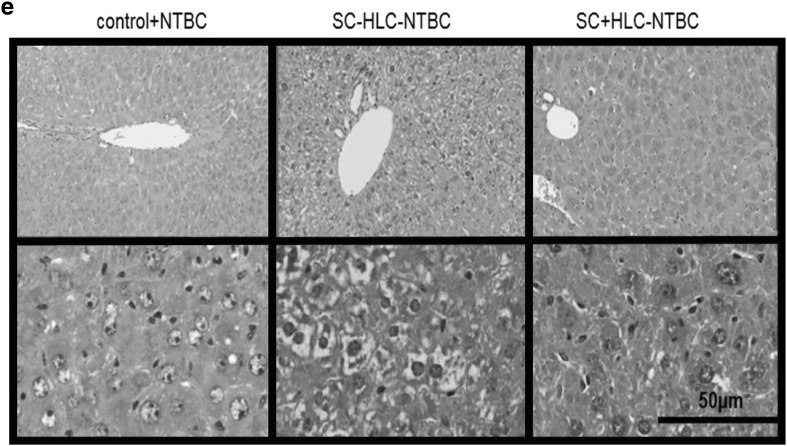
Supportive effect of 3D Hep loaded scaffolds in murine models of tyrosinaemia. **a** Macroscopic images depicting vascularisation on the scaffold (SC, left) and H&E staining showing a slight inflammatory infiltrate and giant cells (GC) within the scaffold (middle), as part of the degradation process. Blood vessels (BV) start to appear as part of the vascularisation process (right, X400). **b** Percentage change in body weight. Conventional FAH knock-out are indicated with open symbols, whereas immunocompromised FAH mice (FRGN) are shown in solid symbols. Circles indicate mice that continued to receive 2-(2-nitro-4-trifluoromethylbenzyol)-1,3cyclohexanedione (NTBC) in both strains of mice. The body weight reduced significantly upon the withdrawal of NTBC in mice receiving acellular scaffolds (squares), although this effect was ameliorated by the presence of HLC in the scaffold (triangles). One-way ANOVA demonstrated that there was a significant difference between transplantation of acellular and cellular scaffolds on the weight of animals between day 8 and day 14 (*p < 0.05, **p < 0.01, ***p < 0.001. ****p < 0.0001). **c** Detectable levels of human albumin were observed in mouse serum with cellular scaffold implantation (solid circles and triangles) but not in the absence of HLC (squares, and open circles); with *p < 0.05, **p < 0.01, indicating that implanted human hepatocytes were functional. **d** Serum levels of markers of hepatocellular damage, ALT, AST, bilirubin and albumin were measured 14 days after implantation of scaffolds; all marker levels were significantly increased after NTBC was replaced with normal drinking water (squares). Open symbols are FAH while solid symbols are FRGN. Markers of liver injury were reduced by the implantation of scaffold with cells, in FAH and FRGN mice. Data were collected from 12 independent experiments with *n* = 3–14 per group. *p < 0.05, **p < 0.01, ***p < 0.001. ****p < 0.0001 by un-paired Mann–Whitney test. **e** Representative images of liver histology are shown indicating liver injury with swollen hepatocytes, apoptotic cells and fat droplets (middle panel). This damage was partially rescued by implantation of the cell containing scaffolds (right panel), with fewer swollen cells and leakage between hepatocytes. This was more similar to control liver histology shown in the left panel

Stem cell-loaded implants were deployed in 2 murine models of tyrosinemia. We successfully transplanted empty scaffolds and 3D Hep-loaded scaffolds into 8- to 12-week-old mice, with wound healing occurring without any infection. The scaffolds were well tolerated and vascularised (Fig. 6a). Three days after transplantation, (2-(2-nitro-4-trifluoromethylbenzyol)-1,3cyclohexanedione (NTBC) was removed from the drinking water in the empty and 3D Hep-loaded scaffolds. Body weight recovery and liver biochemistry was then followed for 14 days. Of note, body weight reduced upon the withdrawal of NTBC in mice receiving acellular scaffolds (squares), while this effect was significantly reduced by the presence of 3D Heps in the scaffold (triangles) between day 8 and day 14 (*p < 0.05, **p < 0.01, ***p < 0.001. ****p < 0.0001) (Fig. 6b). Human albumin was detected in mouse serum with 3D Heps-loaded scaffolds (open and solid triangles) but not in the acellular scaffold groups (squares, and open circles), indicating that the human graft was functional and linked to host circulation (Fig. 6c). Next we studied blood biochemistry. NTBC withdrawal resulted in significant elevation of liver damage markers alanine aminotranferase (ALT), AST and bilirubin (Fig. 6d). This was reduced in the 3D Hep-transplanted group, except for AST in FAH mice and bilirubin in FRGN mice (Fig. 6d). Histological analysis of liver sections was performed from NTBC treated mice (Fig. 6e, left panel), scaffold only treated mice (Fig. 6e, middle panel) and 3D Hep loaded scaffolds (Fig. 6e, right panel). The most severe liver injury was detected in the scaffold only group. Murine livers in this group displayed swollen hepatocytes, apoptotic cells and multi-segmented nuclei (Fig. 6e, middle panel). In addition, there were signs of bile duct alternation. This pathological process was less obvious in the group receiving NTBC (Fig. 6e, left) panel) or 3D Hep-loaded scaffolds(Fig. 6e, right panel).

## Discussion

Mortality associated with end-stage liver disease has increased dramatically over the last 20 years. This is compounded by the shortage of donor organs to treat liver disease. Therefore, scalable tissue models and regenerative therapies are urgently sought. While cell transplantation or bioartificial liver devices offer promise to treat failing liver function, the shortage of good primary human hepatocytes has seriously restricted both endeavours (Demetriou et al. 2004; Francipane et al. 2011; Thompson et al. 2015; Dhawan 2015; Forbes et al. 2015; Oldhafer et al. 2016). Therefore, the establishment of renewable PSC systems has generated much excitement in the field.

Despite significant progress in the field, in vitro derived hepatocyte-like cells generally display a mix of adult and foetal features (Szkolnicka and Hay 2016). To improve cell phenotype, recent research has focussed on 3D differentiation. There have been a number of influential papers published in the 3D liver tissue space. In 2013 Takebe et al. provided proof of concept that mixing primary and stem cell derived cell types led to the generation of liver buds which display improved function in vitro and provided liver support in vivo (Takebe et al. 2013). This was followed by Huch et al. demonstrating that it was possible to isolate, expand and differentiate human bipotent liver progenitors to model human disease (Huch et al. 2015). Later that year the exciting studies by Stevens et al. demonstrated that human primary cell types could be used to build liver aggregates when embedded in a biodegradable hydrogel. Following transplant into mesenteric fat, those aggregates could respond to cues provided by the damaged liver, expanding more than 50-fold in vivo (Stevens 2017). Most recently, pioneering studies from Takebe et al. have shown that it is possible to generate liver buds entirely from human iPSCs and at scale promising a renewable source of human liver tissue (Takebe et al. 2017).

These studies have opened up new experimental avenues for modelling human physiology, regenerative medicine, and gene therapy. While these studies are enabling, the technology described relies on variable components and/or depends on primary human tissue as a source of somatic cells. We believe that this restricts the routine scale up and deployment of such resources. Additionally, the invasive nature of the surgical procedure to deliver to the cells in vivo, combined with testing in immune deficient mice, are limiting factors when translating this technology toward human medicine.

We believe that the construction of stable human tissue under defined conditions, combined with the ability to deploy regenerative therapies in immune competent individuals are important considerations. Therefore, the goal of our studies was to develop a defined differentiation system that was stable in nature and provided liver support in vivo. Notably, 3D Heps demonstrated modest Cyp3A activity for over 1 year in culture providing an exciting opportunity to model human liver physiology and further improve stem cell derived liver tissue. Additionally, stem cell derived tissue provided liver support in vivo and was associated with improved body weight, blood biochemistry and liver histology.

In conclusion, our study demonstrates the importance of 3D differentiation and heterotypic cell interactions to generate stable human liver tissue. Looking ahead, we believe that this study provides a solid foundation with which to build bona fide and renewable liver tissue for human translational medicine.

## Electronic supplementary material

Below is the link to the electronic supplementary material.

Supplementary material 1 (DOCX 22 KB)

Supplementary material 2 (DOCX 3915 KB)

## References

[CR1] Agarwal S, Holton KL, Lanza R (2008). Efficient differentiation of functional hepatocytes from human embryonic stem cells. Stem Cells.

[CR2] Alwahsh SM, Rashidi H, Hay DC (2017). Liver cell therapy: is this the end of the beginning?. Cell Mol Life Sci.

[CR3] Cameron K (2015). recombinant laminins drive the differentiation and self-organization of hESC-derived hepatocytes. Stem Cell Rep.

[CR4] Camp JG (2017). Multilineage communication regulates human liver bud development from pluripotency. Nature.

[CR5] Demetriou AA (2004). Prospective, randomized, multicenter, controlled trial of a bioartificial liver in treating acute liver failure. Ann Surg.

[CR6] Dhawan A (2015). Clinical human hepatocyte transplantation: current status and challenges. Liver Transpl.

[CR7] Ebrahimkhani MR (2014). Bioreactor technologies to support liver function in vitro. Adv Drug Deliv Rev.

[CR8] Forbes SJ, Gupta S, Dhawan A (2015). Cell therapy for liver disease: from liver transplantation to cell factory. J Hepatol.

[CR9] Francipane MG (2011). Management of liver failure: from transplantation to cell-based therapy. Cell Med.

[CR10] Gieseck RL (2014). Maturation of induced pluripotent stem cell derived hepatocytes by 3D-culture. PLoS One.

[CR11] Hannan NR (2013). Production of hepatocyte-like cells from human pluripotent stem cells. Nat Protoc.

[CR12] Hay DC (2007). Direct differentiation of human embryonic stem cells to hepatocyte-like cells exhibiting functional activities. Cloning Stem Cells.

[CR13] Hay DC (2008). Highly efficient differentiation of hESCs to functional hepatic endoderm requires ActivinA and Wnt3a signaling. Proc Natl Acad Sci USA.

[CR14] Hay DC (2008). Efficient differentiation of hepatocytes from human embryonic stem cells exhibiting markers recapitulating liver development in vivo. Stem Cells.

[CR15] Huch M (2013). In vitro expansion of single Lgr5(+) liver stem cells induced by Wnt-driven regeneration. Nature.

[CR16] Huch M (2015). Long-term culture of genome-stable bipotent stem cells from adult human liver. Cell.

[CR17] Loh KM (2014). Efficient endoderm induction from human pluripotent stem cells by logically directing signals controlling lineage bifurcations. Cell Stem Cell.

[CR18] Oldhafer F (2016). Immunological aspects of liver cell transplantation. World J Transplant.

[CR19] Stevens KR et al. (2017) In situ expansion of engineered human liver tissue in a mouse model of chronic liver disease. Sci Transl Med 9(399) 10.1126/scitranslmed.aah5505 PMC5896001 28724577

[CR20] Sullivan GJ (2010). Generation of functional human hepatic endoderm from human induced pluripotent stem cells. Hepatology.

[CR21] Szkolnicka D, Hay DC (2016). Concise review: advances in generating hepatocytes from pluripotent stem cells for translational medicine. Stem Cells.

[CR22] Takayama K (2013). Long-term self-renewal of human ES/iPS-derived hepatoblast-like cells on human laminin 111-coated dishes. Stem Cell Rep.

[CR23] Takebe T (2013). Vascularized and functional human liver from an iPSC-derived organ bud transplant. Nature.

[CR24] Takebe T (2014). Generation of a vascularized and functional human liver from an iPSC-derived organ bud transplant. Nat Protoc.

[CR25] Takebe T (2017). Massive and reproducible production of liver buds entirely from human pluripotent stem cells. Cell Rep.

[CR26] Thompson JA (2015). The effect of extracorporeal C3a cellular therapy in severe alcoholic hepatitis—the Elad trial. Hepatology.

[CR27] Wang Y (2017). Defined and scalable generation of hepatocyte-like cells from human pluripotent stem cells. J Vis Exp.

