# Supplementary material for: 3D human liver tissue from pluripotent stem cells displays stable phenotype in vitro and supports compromised liver function in vivo
Source: Arch Toxicol. 2018 Aug 28;92(10):3117–29. doi: 10.1007/s00204-018-2280-2 (PMC6132688; doi:10.1007/s00204-018-2280-2)
Supplement: Supplementary file 1 — Supplementary material 1 (DOCX 22 KB) [file 204_2018_2280_MOESM1_ESM.docx]

**Supplementary table 1: List of antibodies and optimised dilutions**

**Supplementary table 2: List of antibodies and optimised dilutions**

**Supplementary table 3: List of qPCR primers**
