# Supplementary material for: 3D human liver tissue from pluripotent stem cells displays stable phenotype in vitro and supports compromised liver function in vivo
Source: Arch Toxicol. 2018 Aug 28;92(10):3117–29. doi: 10.1007/s00204-018-2280-2 (PMC6132688; doi:10.1007/s00204-018-2280-2)
Supplement: Supplementary file 2 — Supplementary material 2 (DOCX 3915 KB) [file 204_2018_2280_MOESM2_ESM.docx]

**
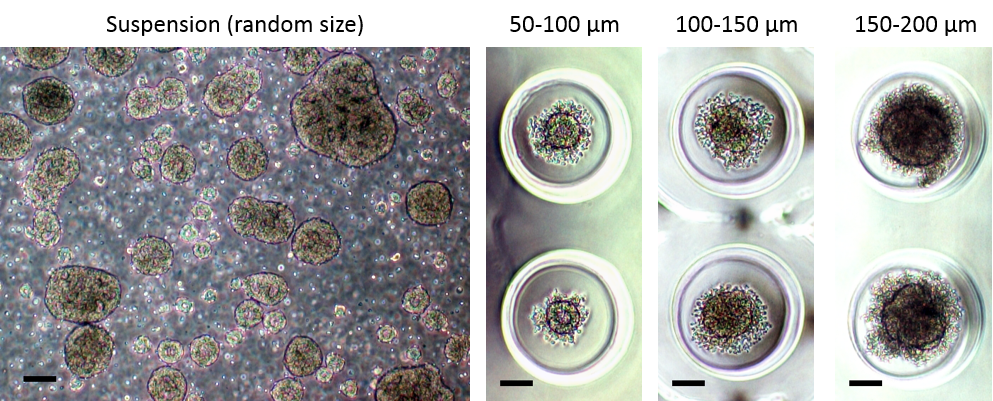


**

**Supplementary figure 1: Generation of 3D Heps.** Self-aggregation in suspension culture resulted in spheroids heterogeneous in size while the initial size of spheroids can be controlled by alteration of seeding density in agarose microplate platform.


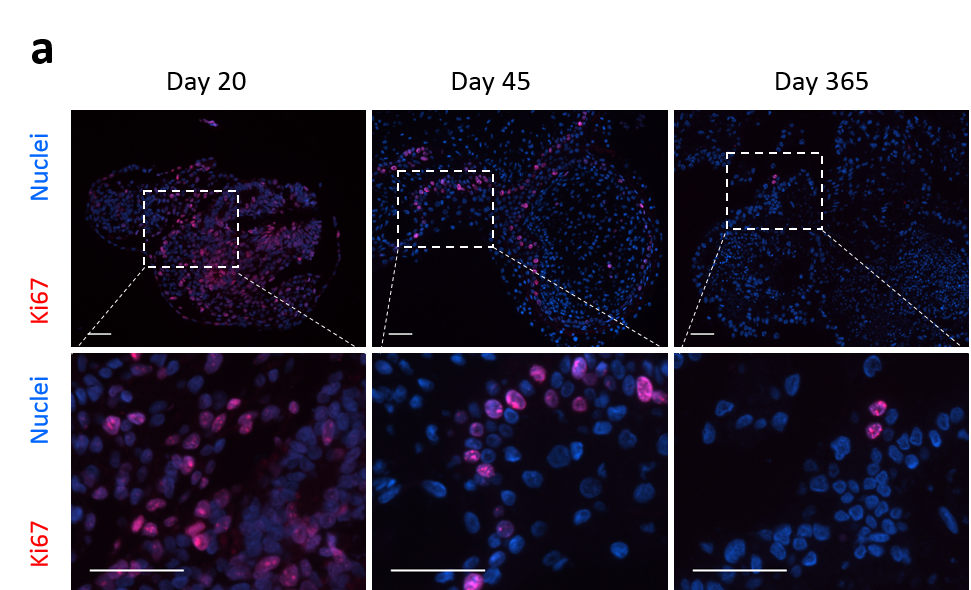

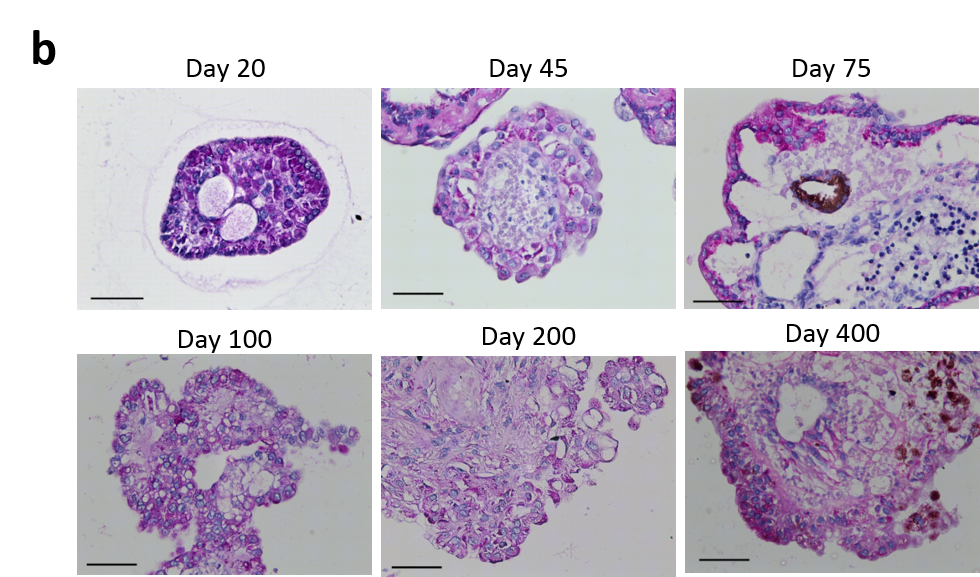


**Supplementary figure 2: 3D Heps characterisation. (a)** The number of proliferative cells reduced by elongation of differentiation. **(b)** Glycogen storage in 3D Heps.


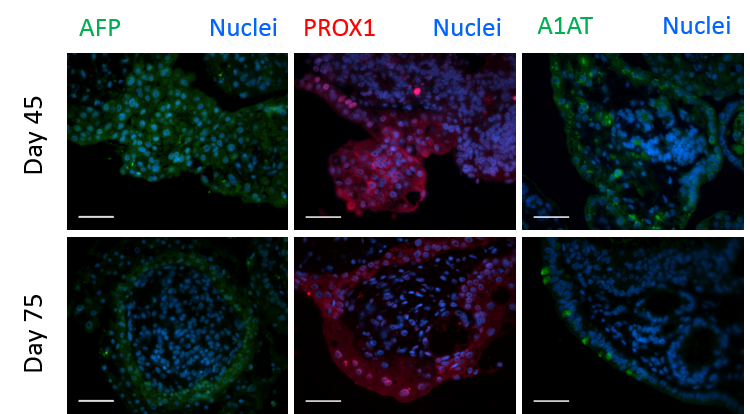


**Supplementary figure 3: Expression of hepatic markers. (a)** AFP expression at d45 and d75. **(b)** Expression of PROX1 at d45 and d75. **(b)** Expression of A1AT at d45 and d75. Scale bar 50 µm.

**
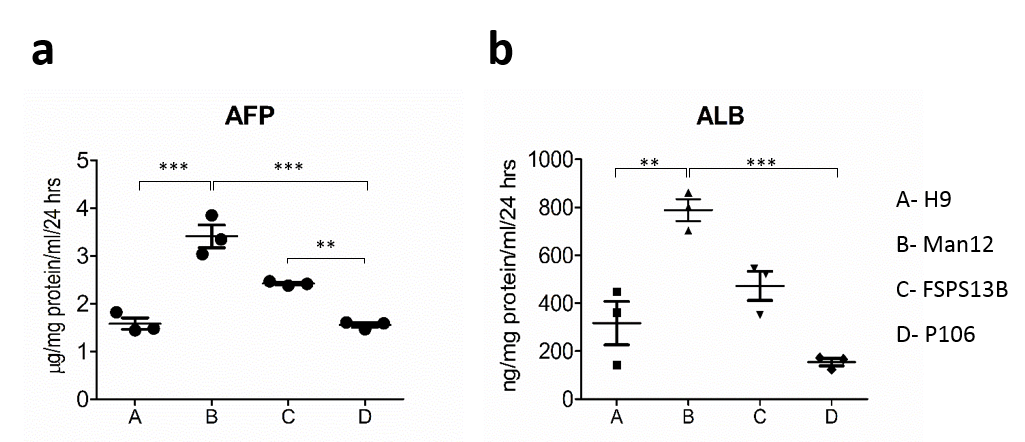
** **
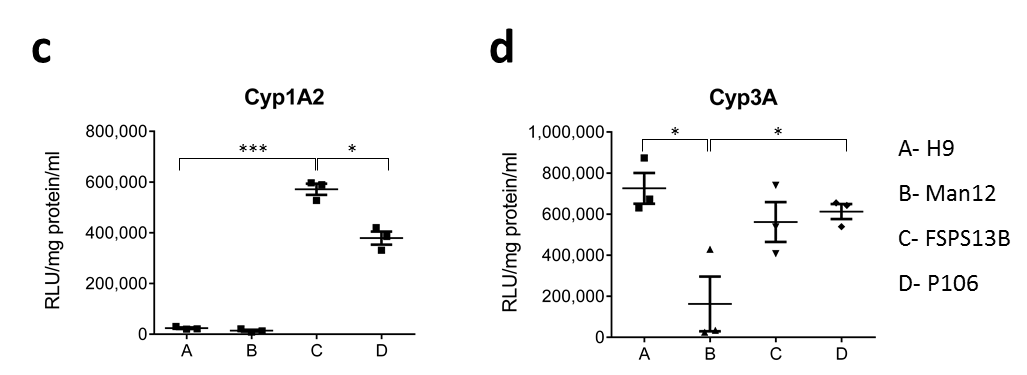
**

**Supplementary Figure 4**: **Functional analysis of 3D Heps generated from two ESC lines (H9 & Man12) and two integration-free iPSC lines (FSPS13B & P106).** **(a)** Secretion of AFP and **(b)** ALB by PSC derived 3D Heps in 24 hrs. **(c)** Cyp1A2 and **(d)** Cyp3A activity detected in PSC derived 3D Heps. n= 3 per group, significance was determined One-way ANOVA Tukey post hoc test. Data are represented as mean ± SEM, * = p<0.05, **=p<0.01, ***=p<0.001.

**
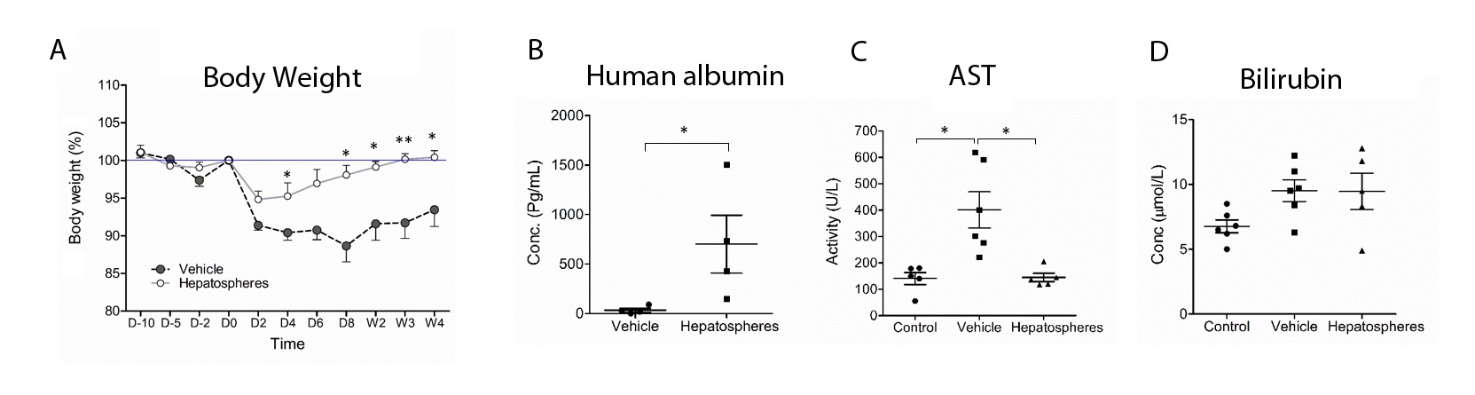
**

**Supplementary Figure 5**: **Stem cell derived hepatocytes provide liver support following 50% partial hepatectomy in immunocompromised mice.**

**(a)** Percentage of body weight change 4 weeks after 50% PHx. Mice weights were measured 10 days before cell transplantation up to 4 weeks post transplantation. **(b)** Human albumin levels in mouse serum after 4 weeks of receiving vehicle or cells, assayed by MSD System. **(c)** Hepatocellular damage markers, aspartate transaminase activity in serum, and **(d)** Serum bilirubin levels after 4 week of 50% PHx. n= 3-7 per group, significance was determined One-way ANOVA Tukey post hoc or by Mann Whitney test, as suitable. Data are represented as mean ± SEM, * = p<0.05, **=p<0.01.
